# Analyzing Depression in College Students Using NLP and Transformer Models: Implications for Career and Educational Counseling

**DOI:** 10.1002/brb3.70828

**Published:** 2025-09-09

**Authors:** Qiuxia Wan, Yue Pan, Sonia Zakeri

**Affiliations:** ^1^ Chengdu Sport University Chengdu China; ^2^ Department of Electrical and Computer Engineering University of Tabriz Tabriz Iran

**Keywords:** college students, depression, educational counseling, natural language processing, social media, transformer models

## Abstract

**Purpose:**

Depression among college students is a growing concern that negatively affects academic performance, emotional well‐being, and career planning. Existing diagnostic methods are often slow, subjective, and inaccessible, underscoring the need for automated systems that can detect depressive symptoms through digital behavior, particularly on social media platforms.

**Method:**

This study proposes a novel natural language processing (NLP) framework that combines a RoBERTa‐based Transformer with gated recurrent unit (GRU) layers and multimodal embeddings. The Transformer captures nuanced language patterns, while the GRU layers account for the sequence of user posts over time. Multimodal embeddings—including behavioral, temporal, and contextual metadata—enhance the model's ability to interpret subtle emotional cues in social media posts.

**Findings:**

The model was evaluated on real‐world datasets from Twitter and Reddit, achieving an accuracy of 90.18% in classifying depressive versus non‐depressive posts. It also demonstrated consistently high performance across both simple and complex sentence types. Statistical comparison with several baseline models confirmed the superiority of the proposed method, particularly over traditional deep learning architectures.

**Conclusion:**

By enabling real‐time detection of depressive signals in social media content, the proposed framework can serve as a practical tool in academic and career counseling. It supports early identification of at‐risk students and facilitates timely interventions, contributing to improved student well‐being, retention, and long‐term success.

## Graphical abstract

A multimodal NLP pipeline uses RoBERTa embeddings with a GRU classifier to detect depression in college students from short posts and interaction signals. The system integrates textual and emoji features and outputs risk levels for early screening and support.

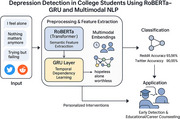

## 1 Introduction

Depression is among the most prevalent mental health disorders, affecting approximately 41.6% of college students, according to recent surveys (Hasib et al. 2023). It has been identified as a leading cause of academic decline, social withdrawal, and even university dropout in several countries (Orabi et al. 2018). Specifically, depression in college populations is associated with decreased academic performance, impaired decision‐making, and increased risk of career disruption. This growing prevalence emphasizes the urgent need for early detection and targeted intervention strategies tailored to this demographic.

With the widespread adoption of social media, students increasingly use platforms such as Twitter and Reddit to articulate personal thoughts, emotional states, and daily experiences. These digital expressions offer a real‐time, high‐volume source of behavioral data that can serve as proxies for mental health monitoring (Bhuvaneswari and Prabha 2023; Anbukkarasi et al. 2023). As social media becomes integral to campus life, it provides a promising avenue for scalable, automated screening of mental health issues—approaches that are faster and more accessible than traditional clinical methods.

Conventional diagnostic techniques, including structured interviews and psychological assessments, remain resource‐intensive and are often inaccessible due to stigma, limited clinical infrastructure, or high costs (Tejaswini et al. 2024; Wani et al. 2022). These constraints have led to growing interest in using natural language processing (NLP) and deep learning to detect signs of depression based on users' linguistic behavior on social media. Earlier models such as convolutional neural networks (CNNs) and long short‐term memory (LSTM) networks have achieved promising results in capturing both semantic content and syntactic structure (Nadeem et al. 2022; Prasad and Jamadagni 2023).

Automated and early detection of depressive symptoms among college students has the potential to transform mental health interventions, academic advising, and career planning. Students suffering from depression frequently demonstrate reduced engagement, poor academic motivation, and indecision regarding future goals. Consequently, intelligent systems capable of identifying early indicators of psychological distress can enable personalized and timely interventions that support both well‐being and educational outcomes (Liu 2024; Wang et al. 2024).

Despite progress in NLP, challenges persist in analyzing social media data due to its informal, noisy nature. These include inconsistent grammar, the use of slang, abbreviations, emojis, and context‐specific language patterns (Mann et al. 2022; Sri et al. 2021). In addition, limited availability of annotated datasets and privacy concerns restrict large‐scale supervised training. Even attention‐based models—though more sophisticated—can be computationally expensive and less adaptable to the nuanced, informal writing often seen in student‐generated content.

NLP plays a critical role in overcoming these challenges by transforming unstructured social media posts into structured representations for deep learning models. Through sentiment analysis, lexical pattern recognition, and temporal modeling, NLP techniques help identify depression‐related indicators such as feelings of hopelessness, isolation, and emotional exhaustion. Embedding techniques like Word2Vec, FastText, and transformer‐based models (e.g., BERT) have improved contextual understanding, enabling more accurate predictions (Garba et al. 2020). Through advanced linguistic and behavioral modeling, the system achieves a high degree of sensitivity to varied sentence structures, emotional tone, and context. This contributes to significantly higher performance compared to previous models (Lara et al. 2021; Cong et al. 2018).

In this study, we propose an NLP‐based framework that combines semantic, temporal, and behavioral insights to detect depressive content in social media posts by college students. The technical architecture—which integrates a Transformer backbone with recurrent layers and multimodal cues—is described in detail in Section 3. This consolidated approach enables the model to interpret both the meaning and evolution of language over time, while remaining sensitive to context, behavior, and sentence complexity.

A key innovation of this work is its sensitivity to sentence complexity, allowing it to adapt to diverse and informal writing styles typically observed in student‐generated content. This adaptability strengthens its applicability for real‐world use in academic settings. Furthermore, the model's high accuracy and interpretability establish it as a strong candidate for integration into early‐intervention frameworks in education and career counseling. The main contributions of this study are summarized as follows:

1. An NLP‐based hybrid framework is introduced for depression detection in college students' social media posts, combining semantic modeling with behavioral and temporal signals.
2. The incorporation of temporal, geographical, and behavioral data enables deeper contextual analysis of student behavior and emotional trends, which has direct implications for supporting educational and career guidance services.
3. The model uniquely classifies and evaluates sentence complexity, enhancing robustness across diverse writing styles and offering improved generalizability for real‐world academic environments.

This paper is organized as follows: Section 2 reviews prior work and its limitations. Section 3 presents the proposed model architecture. Section 4 details dataset selection, preprocessing, and training. Moreover, section 4 presents the evaluation results, including comparisons with existing methods, and further explores the practical implications. Finally, Section 5 concludes with a summary of the findings and outlines future research directions.

## 2 Related Work

Despite the significant impact of depression on individuals and society, research on detecting depressive symptoms through social media analysis remains relatively underexplored. While social media provides a rich source of user‐generated data that could aid in identifying mental health indicators, limited studies have fully leveraged advanced NLP and deep learning models for this purpose. This study aims to address this gap by developing a novel approach that enhances the accuracy and efficiency of depression detection from social media data.

Orabi et al. (2018) developed a deep learning model focused on Twitter to detect depression. Due to the lack of labeled data on Twitter, the model faced challenges, but it leveraged RNN architectures to achieve higher accuracy in depression detection. The primary advantage of this model lies in its simplicity and its ability to utilize semantic features effectively, although challenges remain in handling diverse data.

Anbukkarasi et al. (2023) presented an RNN‐based deep learning model for depression detection from social media data. This model applies CNN and RNN architectures, utilizing feature extraction techniques and processing sparse social media data. Its main advantage is its ability to handle sparse data accurately and extract semantic features, although obtaining labeled data remains a challenge.

Tejaswini et al. (2024) introduced a novel hybrid model named FastText CNN with LSTM (FCL), which incorporates CNNs and LSTM networks for optimized depression detection. The innovation of this model lies in the use of FastText embeddings to enhance text representation, significantly improving model accuracy. However, computational complexity is one of the drawbacks.

Wani et al. (2022) used CNN and LSTM in combination with Word2Vec to detect depression from multi‐source textual data (Twitter, Facebook, and YouTube). Results indicate that the CNN + LSTM hybrid model achieved optimal performance with 99.02% accuracy in detecting depression. The model's use of diverse data sources offers significant advantages, but it is time‐consuming to implement and optimize.

Nadeem et al. (2022) developed a model for depression detection by combining CNN and LSTM architectures with an attention mechanism to analyze Twitter data across two labeled categories. The SSCL model achieved impressive results with 97.4% accuracy for binary‐labeled data and 82.9% accuracy for three‐class labeled data, outperforming previous models in terms of accuracy.

Prasad and Jamadagni (2023) used social media data to detect depression. The proposed model combines bidirectional LSTM and CNN for feature extraction and context learning, achieving an accuracy of approximately 93% in depression detection. This AI and machine learning‐based system effectively identifies depression symptoms, although additional development is needed for audio and video data processing.

Mann et al. (2022) proposed a multiple‐instance learning approach for depression detection from social media data. This model utilizes Transformer and LSTM architectures, tested on data from Brazilian students. With its focus on analyzing individual posts to detect user‐level depression, the model achieves higher accuracy than similar methods. Due to its multi‐instance learning approach, the model offers greater interpretability, though its implementation is complex.

Sri et al. (2021) focused on identifying depression among users in various Malaysian cities by analyzing sentiments in their tweets. Using LSTM as the core architecture, the model was able to achieve around 94% accuracy in detecting depression, which was especially useful during the COVID‐19 pandemic, though optimization for local languages and additional data remain challenges.

Bhog et al. (2022) employed the BERT algorithm to analyze social media texts for depression detection. This approach processes user data through multi‐stage categorization to identify depression at different stages. The main innovation is the use of multiple learning models to label user posts, enhancing detection accuracy, though the model's computational complexity presents a challenge.

F. M. Shah et al. (2020) developed a hybrid model combining BiLSTM and machine learning to detect depression using textual data from social media. The proposed model can identify depression in processed Reddit data, utilizing feature extraction techniques to improve accuracy. Its main strength is its high performance and ability to analyze diverse data, though parameter optimization is a primary challenge.

Kour and Gupta (2022) used a CNN‐LSTM combination with Word2Vec feature extraction techniques to detect depressive sentiment on Twitter. This deep learning‐based method provides higher accuracy compared to traditional machine learning models. The main advantage lies in its use of prominent linguistic features to improve depression detection accuracy, though computational complexity and cost pose challenges.

Cui et al. (2022) employed an attention‐based reinforcement network to extract deep emotional features from users for depression detection. The proposed model focuses specifically on detecting depression‐indicative posts, achieving high precision and F1 scores. Its main strength is the ability to extract deep semantic features and use attention networks to improve accuracy, though it requires significant optimization.

Wongkoblap et al. (2021) explored a deep learning model with anaphora resolution capabilities for identifying users with depression on Twitter. This model utilizes semantic features in user posts to detect depression and can recognize mental health‐related content. It excels in accurately identifying language that reflects psychological states, though the model requires labeled data to optimize parameters effectively.

Kabir et al. (2022) explored depression detection in Bengali texts, developing a unique corpus labeled with input from mental health experts. Using machine learning models like kernel SVM and deep learning techniques such as LSTM and gated recurrent units (GRU), they achieved high accuracy, with GRUs reaching 81%. Their work highlights the potential of NLP and deep learning for mental health assessment, despite data limitations in Bengali.

Zogan et al. (2022) tackled the issue of explainability in depression detection by developing a hierarchical attention‐based model (MDHAN) that interprets predictions. By applying tweet‐ and word‐level attention, MDHAN assesses the importance of each word and tweet, capturing patterns across user timelines. Their results show that MDHAN not only improves accuracy in detecting depression but also provides clear evidence behind its predictions.

Similar to these solutions, recent models by researchers such as Marriwala et al. (Marriwala and Chaudhary 2023), Kour and Gupta (2022), Amanat et al. (2022), Ghosh et al. (2023), Malhotra and Jindal (2022), Shabana and Bharathi (2024), Khan and Alqahtani (2024), and Beniwal and Saraswat (2024) have been developed, with several prominently incorporating NLP techniques.

Existing methods for depression detection from social media, while leveraging NLP and deep learning, often struggle with issues like limited labeled data, high computational costs, and challenges in handling diverse, noisy datasets. Many of these models, though accurate, also lack interpretability and require significant optimization for multilingual contexts (Hasib et al. 2023; Wani et al. 2022; Santos et al. 2023; Bokolo and Liu 2024).

Qasim et al. (2025) utilized Sentence Transformers and advanced transformer‐based models to classify depression severity (mild, moderate, or severe) in Reddit posts. They emphasized the applicability of these techniques in scalable early intervention and mental health monitoring systems.

Recent advances in BERT‐based models have extended their utility beyond classification tasks into domains such as extractive summarization, demonstrating improved contextual representation and information retention. For example, the work by Bano and Khalid (2022) proposed a novel BERT‐based architecture for scholarly text summarization, highlighting the model's adaptability to long‐form semantic understanding (Bano and Khalid 2022). These architectural refinements and applications underscore the potential of transformer‐based models in capturing complex linguistic patterns, which is particularly valuable in nuanced tasks like mental health detection from social media content.

Kokane et al. (2024) evaluated four transformer models—BERT, XLNet, RoBERTa, and DistilBERT—on Twitter and Reddit datasets and concluded that these models accurately detect signs of depression. Their findings suggest these models serve as effective tools for assessing mental health conditions in online environments.

Raj et al. (2024) focused specifically on the BERT model for detecting depression in Reddit posts, achieving high accuracy and an F1‐score exceeding 91%. They proposed the model as a reliable solution for early digital mental health interventions.

Ji et al. (2023) developed a hybrid model combining EmoBERTa with a transformer architecture, incorporating both textual content and user behavioral features such as posting time and tweet content. The resulting multimodal model closely aligns with the approach of integrating multimodal embeddings used in this study.

Pandey and Kumar (2024) demonstrated that transformer‐based models—particularly RoBERTa—are highly effective in predicting stress and depression from social media posts. Their work highlights the predictive power of NLP for improving mental health services through early detection.

A recent study (S. M. Shah et al. 2025) demonstrated that fine‐tuned large language models such as GPT‐3.5 Turbo and LLaMA2‐7B can detect depressive content on social media with 96.4% accuracy, outperforming baseline and state‐of‐the‐art models, highlighting their potential for early depression diagnosis across platforms.

Moreover, Bagroy et al. (2017) analyzed user‐generated content from over 100 university‐specific Reddit communities and developed a model that achieved 97% accuracy in detecting mental health expressions. Their findings indicate that the linguistic patterns in these forums reflect broader trends in student mental well‐being, leading to the creation of a “Mental Well‐being Index” for each campus.

Suhail et al. (2024) conducted a comparative analysis of Reddit and Twitter posts related to depression and anxiety. The study emphasized that users on both platforms express mental health concerns in similar ways, revealing shared linguistic features that are applicable to specific populations such as college students.

D'Cruz et al. (2023) utilized combined Reddit and Twitter datasets to predict depression and highlighted that the language used on these platforms effectively captures users' psychological states, even when the data are not explicitly sourced from student populations.

The proposed model addresses these limitations by integrating Transformer models like RoBERTa with a GRU layer and multimodal embeddings, enhancing both the accuracy and interpretability of depression detection. Leveraging multi‐head attention within the Transformer, along with optimized embeddings that capture temporal, geographical, and behavioral information, the model improves data sparsity handling and reduces computational demands. This design results in a scalable and efficient solution, ideally suited for diverse, real‐world social media data.

## 3 Proposed Method

The proposed method combines Transformer models, specifically RoBERTa, with a GRU layer and multimodal embeddings to improve accuracy and interpretability in detecting depression from social media texts. By leveraging multi‐head attention in the Transformer, the model captures deep linguistic and semantic features, while the GRU layer processes temporal dependencies, enhancing analytical precision. The addition of multimodal embeddings—incorporating temporal, geographical, and behavioral information—enables a more comprehensive understanding of the social context related to depressive behaviors, making the method robust for real‐world social media data.

### 3.1 Model Architecture Overview

This section outlines the proposed framework's core components, including the integration of RoBERTa for deep semantic analysis, GRU layers for processing temporal dependencies, and multimodal embeddings for capturing contextual information. This architecture is designed to effectively analyze and classify depressive language patterns within social media text, ensuring a high level of accuracy and adaptability.

#### 3.1.1 Transformer Component

The Transformer component of the proposed model employs RoBERTa, a variant of the BERT architecture optimized for robust text encoding. RoBERTa (Robustly Optimized BERT Pretraining Approach) builds on the original BERT model by removing the Next Sentence Prediction objective and incorporating a dynamic masking technique, which enhances its ability to capture deeper semantic nuances in language. This feature is particularly valuable for depression detection from social media texts, where nuanced language and context are critical. In RoBERTa, multi‐head self‐attention mechanisms allow the model to weigh the importance of words in a sentence differently based on context, capturing dependencies between words regardless of their distance within the text.

**FIGURE 1:**
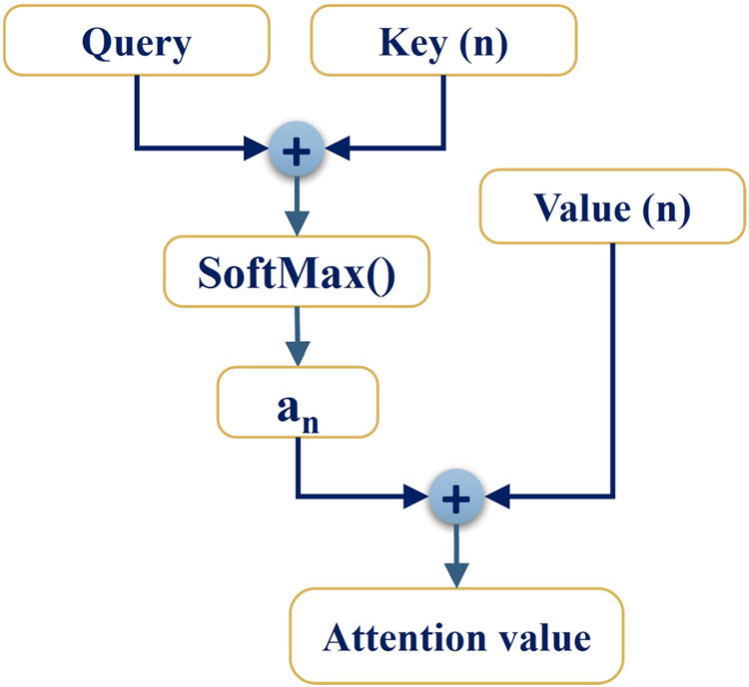
The figure illustrates the process of calculating attention.

The core of the attention mechanism involves redistributing weights and doing a weighted summation of “Value” based on “Key” and “Query.” From a formal standpoint, the attention procedure may be likened to a value–key–query that links key‐values and input queries to produce an output. Moreover, the weights represent the degree of similarity between “Key” and “Query,” and the output is the result of multiplying each “Value” by its corresponding weight (Suhail et al. 2024). The dimensions “Value” (*d*_v_) and “Key” (*d*_k_) are inputted into the architecture, as seen in Figure 1.

The similarity between “Key” and “Query” is determined by dividing a dot product function by (*d*_k_)^0.5^. The weights of each “Value” are obtained by the utilization of the softmax method. The “Key” dimension (*d*_k_) represents a set of input queries that are to be matched against the key‐value pairs in the network. The “Value” dimension (*d*_v_) represents the corresponding output for each query. The attention mechanism works by computing the degree of similarity between the “Query” and “Key” dimensions and redistributing weights to produce an output by multiplying each “Value” by its corresponding weight. The attention function in a neural network allows it to focus on specific aspects of the input information. By consolidating the “Value,” “Key,” and “Query” into matrices, the attention function can calculate weighted connections between different data points, enabling it to highlight the most important information and make accurate predictions or recommendations. The attention function is effectively executed by consolidating “Value,” “Key,” and “Query” into matrices V, K, and Q. A particular “Query” is employed to ascertain the level of similarity among the “Keys” and multiple “Query,” and for each “Value,” the weight coefficient of each “Key” is calculated. The dot product operates in accordance with the following rules:

(1) $$S_{i}\left(Q,K_{i}\right)={\left(d_{k}\right)}^{-0.5}\times K_{i}^{T}\times Q$$

In this equation, the scale factor is (*d*_k_)^−0.5^, and its main purpose is to alter the calculation result. In order to complete and standardize the numerical conversion of the original score, one might employ a classifier known as softmax. The initially computed score will be converted into a likelihood distribution by aggregating all element weights. Simultaneously, the intrinsic mechanism of the softmax classifier enables it to adapt the weights for increasingly influential elements. The softmax function (SoMax) output, *a_i_*, is computed using the following:

(2) $$a_{i}=SoMax\left(S_{i}\right)=\exp^{S_{i}}\times {\left(\sum_{i=1}^{n}exp^{S_{i}}\right)}^{-1}$$

Moreover, an effective expression for representing scaled dot‐product attention is as follows:

(3) $$Atn\;\left(Q,K_{i},V_{i}\right)=V_{i}\times SoMax\left(K_{i}^{T}\times Q\times {\left(d_{k}\right)}^{-0.5}\right)$$

Initial comparisons are made between “Key” and “Query” to determine whether they are related to dot products. Next, the SoMax is applied to derive the weight coefficient. Finally, the “Value” is calculated by taking the weighted sum and multiplying it by the weight coefficient. We have now completed the method for calculating attention mechanisms. This self‐attention mechanism allows RoBERTa to assign varying levels of significance to each word in a given sentence based on its context, capturing the intricate linguistic features needed to identify patterns associated with depression.

#### 3.1.2 Mechanism for Multi‐Head Attention

**FIGURE 2:**
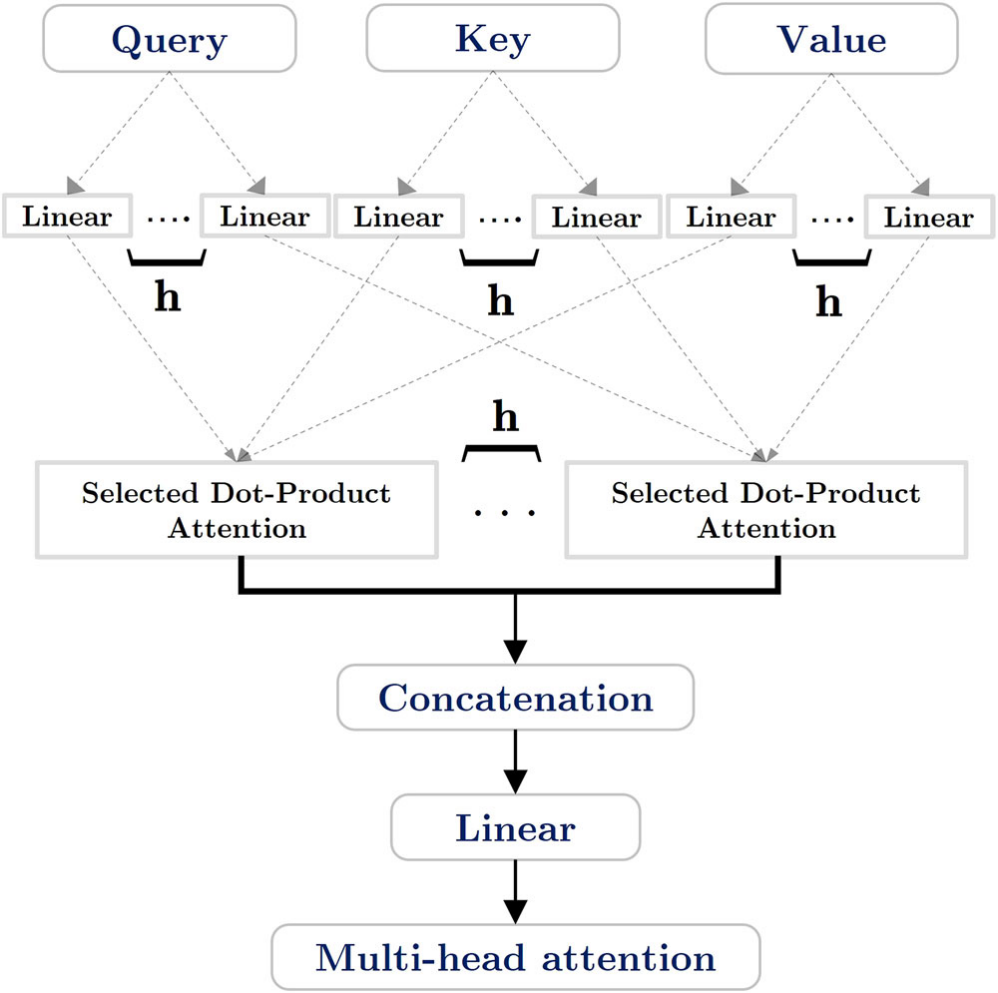
An attention mechanism with multiple heads is illustrated in this figure.

Notably, the multi‐head attention procedure is a distinctive approach to computing attention, employing scaled dot products. The model enables the multi‐head attention mechanism to acquire several mapping functions, as seen in Figure 2. The linear transformation is applied to all three *d*_models_ (“Value,” “Key,” and “Query”), and subsequently the scaled dot product attention is computed simultaneously to get an output with *d*_v_ dimensions. The final output value is achieved by applying a linear transformation to the integrated outputs of the *Concatenation* function, which are repeated ℎ times.

The multi‐head attention strategy enhances the model's perceptual capabilities by enabling it to learn the correlation data in separate depiction subspaces, without introducing additional complexity to the algorithm. Below is the mathematical formula for calculation:

(4) $${\mathrm{Multi}}_{\mathrm{head}}\left(Q,K_{i},V_{i}\right)=\mathrm{concatenate}\left({\mathrm{head}}_{1},{\mathrm{head}}_{2},\cdots ,{\mathrm{head}}_{h}\right)W^{O}$$

where,

(5) $${\mathrm{head}}_{i}=\mathrm{atn}\left({\mathrm{QW}}_{i}^{Q},{\mathrm{KW}}_{i}^{K},{\mathrm{VW}}_{i}^{V}\right)$$

moreover,

(6) $$W≔\left\{\begin{array}{c} W_{i}^{Q}\in R^{d_{\;\mathrm{mod}\;el}\times d_{k}}\\ W_{i}^{K}\in R^{d_{\;\mathrm{mod}\;el}\times d_{k}}\\ W_{i}^{V}\in R^{d_{\;\mathrm{mod}\;el}\times d_{v}}\\ W^{O}\in R^{hd_{v}} \end{array}\right.$$

The existence of eight parallel attention levels in this research implies that *h* is equal to 8. The values of *d*_k_, *d*_v_, and *d*_model_ */h* are equal to 64, where *h* represents the divisor. The total computational cost is similar to that of the full‐dimensional single‐head attention layer, as the dimensionality of each attention layer decreases.

The multi‐head attention strategy is less complex than the scaled dot‐product attention method, allowing the model to acquire diverse representational information while minimizing loss of target information through averaging. Moreover, in the multi‐head attention setup, multiple self‐attention heads operate in parallel, with each head focusing on different aspects of the sentence. These heads are later concatenated, allowing the model to derive a richer representation of the text. The output of multi‐head attention is then fed through a residual connection and layer normalization, enhancing the stability and expressiveness of the model. This process is repeated across multiple layers, enabling RoBERTa to extract robust, context‐sensitive features from social media text, essential for the accurate detection of depressive language patterns.

#### 3.1.3 Gated Recurrent Unit

**FIGURE 3:**
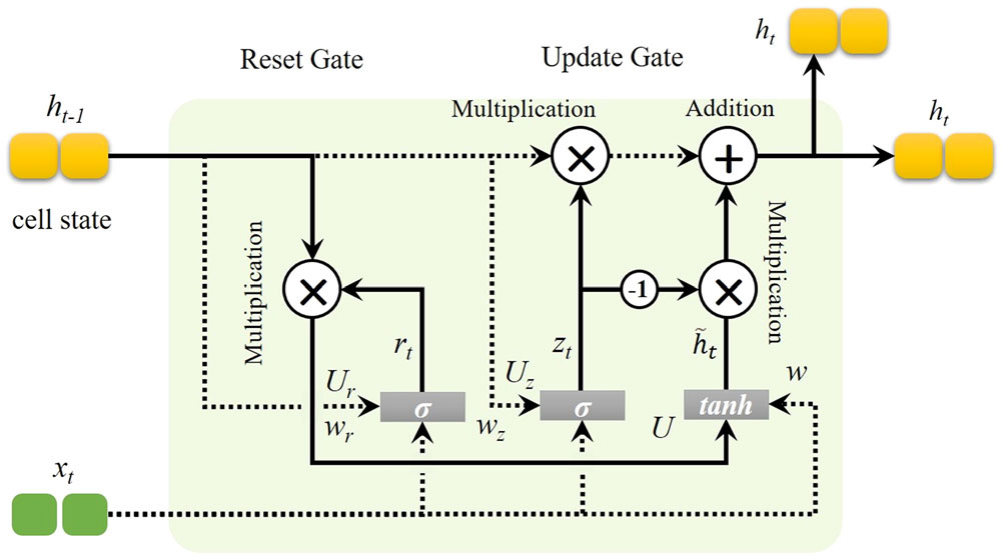
Architecture of the GRU Cell, which processes temporal dependencies within the proposed depression detection model. Positioned after the Transformer layer, the GRU captures sequential patterns in social media text, enhancing the model's ability to classify depressive indicators accurately by retaining context across user posts.

The GRU layer in the proposed model plays a crucial role in capturing temporal dependencies within the text data, enhancing the model's ability to identify sequential patterns associated with depressive expressions. While transformer models like RoBERTa excel at understanding context through attention mechanisms, GRUs are particularly effective in processing sequential information by maintaining a memory of previous inputs, making them valuable for time‐dependent text analysis in social media. As shown in Figure 3, the GRU operates through two primary gates: the update gate (z_𝑡_) and the reset gate (𝑟_t_). These gates control the flow of information and determine how much of the past information to retain or discard, allowing the model to focus on relevant historical context for each time step. The update gate and reset gate are computed as follows:

(7) $$z_{t}=\sigma \left(w_{z}\cdot \left[h_{t-1},x_{t}\right]\right)$$

(8) $$r_{t}=\sigma \left(w_{r}\cdot \left[h_{t-1},x_{t}\right]\right)$$

where *σ* represents the sigmoid activation function, *w_z_* and *w_r_* are weight matrices for the update and reset gates, respectively, *h_t−1_* is the previous hidden state, and *x_t_* is the current input from the Transformer's output.

The reset gate, *r_t_*, controls the amount of previous information to forget, while the update gate, *z_t_*, decides how much of the new information to keep. The hidden state (*h_t_*) at the current time step is updated as follows:

(9) $${\tilde{h}}_{t}=\tanh\left(W\cdot \left[r_{t}*h_{t-1},x_{t}\right]\right)$$

(10) $$h_{t}=\left(1-z_{t}\right)*h_{t-1}+z_{t}*{\tilde{h}}_{t}$$

here, ${\tilde{h}}_{t}$ represents the candidate hidden state, ⁕ denotes element‐wise multiplication, and *h_t_* ​is the final hidden state for the current time step.

The GRU layer receives the contextual embeddings from the Transformer and processes these sequentially, enabling the model to retain relevant patterns across posts or sentences. This ability to model temporal dependencies complements the Transformer's contextual embeddings, resulting in a richer, more nuanced understanding of depressive language patterns. By combining the Transformer's deep semantic features with the GRU's time‐sensitive processing, the model improves its overall accuracy and depth of analysis in detecting signs of depression.

#### 3.1.4 Multimodal Embeddings

In this section, we discuss the use of multimodal embeddings to enhance the model's understanding of social context in detecting depression. Multimodal embeddings integrate various types of data beyond plain text, such as temporal information (e.g., the timing and frequency of posts), geographical data (e.g., location‐based trends in language use), and behavioral patterns (e.g., interaction levels or posting habits). These additional layers of data enable the model to capture nuanced signals associated with depressive behaviors that may not be apparent from text alone. To incorporate multimodal embeddings, we process each data type separately, creating a unique embedding for each modality. For example, temporal embeddings are generated by encoding time‐based features, while geographical embeddings capture location‐specific variations in language. These embeddings are then concatenated with the main text embeddings to form a comprehensive representation of each user's posts. By integrating these multimodal features, the model can better understand the complex social and emotional contexts underlying user behaviors, resulting in a more accurate and context‐sensitive depression detection framework.

### 3.2 Role of NLP in Depression Detection

In our proposed model, RoBERTa is the foundation for NLP, enabling precise extraction of semantic features specifically linked to depressive language patterns. Trained on vast datasets, RoBERTa captures nuanced meanings and context‐dependent expressions within social media text, which are critical in identifying words and phrases that reflect negative emotions and hopelessness—hallmarks of depressive speech. By providing deep semantic understanding, RoBERTa allows our model to go beyond surface‐level keywords, capturing complex emotional contexts vital for accurate classification.

RoBERTa's ability to generate contextualized embeddings allows for more effective sentiment analysis, a critical component in detecting depression. Individuals with depression often use language conveying sadness, frustration, or isolation, and these sentiments may not always be overtly expressed. Through its contextual embeddings, RoBERTa enables our model to identify these subtle emotional tones within posts, enhancing the model's sensitivity to underlying depressive indicators and improving its ability to detect nuanced expressions of distress.

Table 1 outlines the various roles RoBERTa performs in the proposed model, detailing its objectives, processing methods, benefits, and examples of specific depressive indicators detected. Another advantage of RoBERTa within our NLP framework is its capability to identify structural language patterns unique to individuals experiencing depression. Depressed individuals may frequently use simpler, repetitive sentence structures and display a monotonous tone, reflective of their mental state. RoBERTa's architecture effectively analyzes these structures, enabling our model to detect not only specific depressive terms but also these deeper, structural markers of depression. This layer of analysis allows the model to recognize depression‐related language patterns that go beyond individual word choices.

**TABLE 1:** Roles and objectives of RoBERTa in language analysis for depression detection.

Role of RoBERTa	Objective	Processing method	Benefit	Examples of detected indicators
Semantic feature extraction	Identify words and phrases associated with depression, such as feelings of hopelessness or worthlessness.	Word embeddings, multi‐head attention	Captures deeper meanings beyond surface words	Keywords like "hopeless," and "alone," "worthless"
Sentiment analysis	Detect negative or melancholic tone in user posts, enhancing sensitivity to underlying depressive cues.	Contextual embeddings, sentiment scoring	Improves detection of nuanced emotional expressions	Phrases reflecting sadness, frustration, or apathy
Identification of depression‐specific language structures	Recognize structural language patterns (e.g., short sentences, repetitive tone) commonly used by individuals with depression.	Sequence analysis, grammar pattern recognition	Identifies linguistic patterns that indicate depressive states	Simplified syntax, repetitive phrasing, monotone expressions
Contextual understanding of user interactions	Analyze language within the context of user history and social interaction patterns.	Multi‐level attention, temporal sequencing	Provides context‐sensitive insights on depression over time	Changes in tone or expression frequency across posts
Integration with multimodal data	Combine textual analysis with temporal, geographical, and behavioral features for richer context.	Cross‐modality embeddings, feature fusion	Enhances understanding of social and situational context	Correlation of depressive tone with time, location, and behavior

Our choice of RoBERTa as the NLP component in this model is based on its proficiency in capturing detailed, context‐sensitive representations that are essential for accurately detecting depression. RoBERTa's transformer‐based structure enables the model to understand and interpret complex, real‐world social media language with precision. By incorporating RoBERTa, our approach leverages the full power of NLP to handle the intricate nature of depressive language on social media, resulting in a more reliable and accurate depression detection process.

### 3.3 Data Preprocessing and Feature Extraction

Data preprocessing is a critical step in preparing social media text data for accurate and efficient depression detection. Given the unstructured and often noisy nature of social media posts, preprocessing focuses on removing irrelevant elements (e.g., typos, special characters, excessive punctuation) and optimizing the text for analysis. The goal is to standardize the data, enhancing its readability and consistency for the model. This process allows the depression detection model to focus on meaningful language patterns while minimizing the impact of distracting or irrelevant noise in the data.

Data cleaning and normalization ensure that the text input aligns with a standard format, ready for model training. This process includes converting text to lowercase, removing stop words, handling slang, and standardizing abbreviations to achieve consistency across the dataset. Additionally, techniques like lemmatization and stemming are applied to reduce words to their base or root forms, helping the model interpret words with similar meanings more effectively. By implementing these cleaning and normalization steps, we create a streamlined dataset that facilitates improved feature extraction and reduces computational demands.

Embedding feature selection plays a vital role in capturing the most relevant aspects of the text and multimodal data for accurate depression detection. Using NLP embeddings, such as word embeddings or contextual embeddings, we transform the text data into numerical vectors that the model can process. Additionally, multimodal features—such as temporal, geographical, or behavioral information—are integrated to enrich the context. By carefully selecting and embedding these features, the model gains a nuanced understanding of both language and contextual data, leading to more precise and context‐aware predictions of depressive indicators.

## 4 Results

In the Results section, we evaluate the model's performance using metrics such as accuracy, precision, recall, and F1‐score, comparing it with baseline methods. This section highlights the model's improvements in detecting depression‐related language and the benefits of multimodal embeddings and sequential analysis in enhancing predictive accuracy.

### 4.1 Setting

For the system configuration, the model was trained and evaluated on a setup with an Intel i5 processor, which provided sufficient computational power for handling the moderate requirements of our hybrid deep learning architecture. Development was carried out using Python, with libraries like Gensim and NLTK (Vaswani et al. 2017) for data preprocessing and embeddings. The core model components, including the Transformer‐based RoBERTa layer and the GRU layer, were implemented and trained using TensorFlow and Keras (Chicho and Sallow 2021; Haghighat and Juanes 2021), which enabled smooth model construction and training management. To ensure optimal model performance, we configured the hyperparameters with a training schedule of 20 epochs and a dropout rate of 0.25 for regularization. The Adam optimizer was selected, given its adaptability with learning rates and efficiency in convergence, making it a robust choice for depression detection tasks. These configurations were designed to support effective model training and testing while meeting the computational requirements of the proposed architecture.

The training process for this depression detection model was structured to optimize learning efficiency and performance stability. A batch size of 32 was chosen to balance training speed with memory efficiency, allowing the model to update weights frequently across each epoch. The training spanned 20 epochs, which proved sufficient for convergence without overfitting. Throughout the training, we maintained a dropout rate of 0.25 to prevent overfitting, and weights were updated after each batch to ensure that the model effectively captured linguistic patterns associated with depression.

Hyperparameter tuning was centered around the optimal configuration of learning rates, attention heads, and units in the RoBERTa and GRU layers. The learning rate was set at 0.001 using the Adam optimizer for its stability and adaptive capabilities. The Transformer component, RoBERTa, incorporated 12 attention heads to capture diverse contextual dependencies, while the GRU component utilized two layers to efficiently model temporal dependencies. Dropout was consistently set at 0.25 during tuning to maintain robust regularization across layers, enabling the model to learn depressive language patterns accurately while retaining generalization capacity.

### 4.2 Dataset

The datasets employed in this study were sourced from two prominent social media platforms: Twitter (Amanat et al. 2022) and Reddit (Joseph et al. 2021). The Reddit dataset comprises approximately 13,000 posts, evenly balanced between 6500 labeled as “depressive” and 6500 as “non‐depressive” (see Table 2). The Twitter dataset, obtained from Kaggle, contains 6164 posts, also balanced between depressive and non‐depressive categories. Although these datasets are not explicitly composed of posts made solely by college students, prior empirical research provides strong evidence that linguistic and behavioral patterns on these platforms closely align with those commonly observed in student populations. Bagroy et al. (2017) developed a social media‐based index for monitoring mental well‐being specifically on college campuses, identifying characteristic language markers. Suhail et al. (2024) conducted a comparative sentiment analysis on mental health‐related posts from Reddit and Twitter, focusing on platform‐specific linguistic features prevalent among younger users, including students. Furthermore, D'Cruz et al. (2023) demonstrated the utility of combined Reddit and Twitter data for depression prediction within contexts closely related to student experiences.

**TABLE 2:** Overview of the datasets used for depression detection from social media posts.

Platform	Total posts	Depressive posts	Non‐depressive posts	Source
Reddit	13,000	6500	6500	Original Reddit data
Twitter	6164	3082	3082	Kaggle

In addition to leveraging these empirical studies, our data collection process included keyword filtering targeting academic stress, social isolation, and mental health‐related terms, which are prevalent concerns within college student communities. This methodological step further ensures that the dataset is reflective of the demographic under study. Nonetheless, it is important to note that the absence of verified college student identities remains a limitation. Future research should seek datasets with confirmed student participants to enhance the specificity and validity of these findings. Both datasets underwent extensive preprocessing, including tokenization, normalization, and removal of noise, to optimize feature extraction and classification performance. Furthermore, both Reddit and Twitter have been shown to be widely used by college students for expressing emotional distress, academic pressure, and personal struggles. These platforms provide semi‐anonymous environments that are conducive to open discussions about mental health, particularly among young adults. According to recent usage statistics and behavioral studies, a significant portion of social media mental health discourse originates from individuals within the traditional college‐age demographic (18–24 years), making these sources especially relevant for modeling depression‐related language in student contexts. Thus, even in the absence of explicit demographic tagging, the content itself—combined with platform‐specific user behaviors—offers a reliable approximation of student mental health expressions in real‐world settings.

### 4.3 Evaluations

**FIGURE 4:**
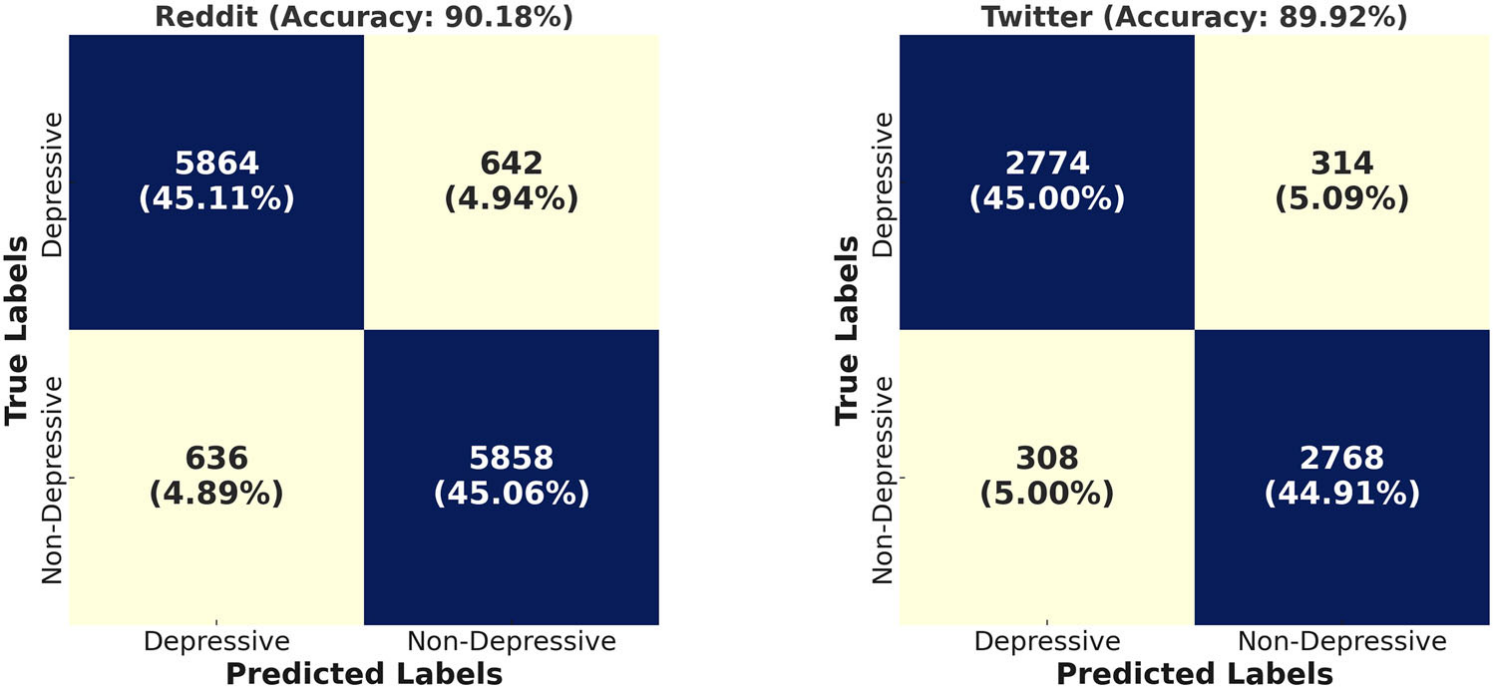
Confusion matrices illustrating the classification performance of the proposed model on depressive and non‐depressive posts across the Reddit and Twitter datasets.

In the initial stages of experimentation, our primary focus was to classify depressive and non‐depressive posts across two distinct datasets: Twitter and Reddit. To assess the model's effectiveness, we computed confusion matrices, which are shown in Figure 4. These matrices allow for a clear visualization of the distribution of classifications in each category on both platforms, showcasing the precision and recall rates achieved. For the Reddit dataset, an accuracy of 90.18% was obtained, while the Twitter dataset demonstrated slightly lower performance, with an accuracy of 89.92%. These preliminary results suggest that our proposed method is robust across both datasets, yet there is a marginal decrease in performance with the Twitter data, likely attributable to dataset‐specific features.

The confusion matrices reveal that the model efficiently distinguishes depressive content from non‐depressive content, with a precise balance between false positives and false negatives across both platforms. The minor variations in classification errors are proportionate to the dataset sizes, and these results illustrate the model's adaptability. Particularly, the model displays a sensitivity to underlying depressive language patterns, which is essential for accurate detection in the nuanced context of social media. This sensitivity indicates the model's strength in addressing subtle, often hidden signs of depression, which are commonly present in user‐generated posts but may not follow standard linguistic patterns. The Reddit dataset exhibits more stable classification outcomes due to its larger volume of posts, which allows the model to adjust more accurately to language patterns. The results suggest that the model can leverage the more varied linguistic structures of Reddit posts, showcasing robustness and in‐depth semantic analysis. The incorporation of multi‐head attention mechanisms and GRU layers further contributes to this stability by allowing the model to identify and retain long‐term dependencies—crucial for understanding the sequential nature of depressive expressions over time. This combination highlights the model's robustness in dealing with unstructured, dynamic data, particularly valuable for analyzing large‐scale social media datasets like Reddit.

The model demonstrates high accuracy in distinguishing depressive from non‐depressive posts, achieving reliable classification across platforms by capturing nuanced linguistic and structural features essential for identifying depressive cues. Additionally, the integration of GRU layers with RoBERTa equips the model to process time‐dependent information, capturing patterns that develop across multiple posts or interactions, thereby adding depth to the analysis of sequential behaviors. Leveraging RoBERTa's Transformer architecture further enhances the model's capacity for deep semantic understanding, allowing it to recognize complex contextual meanings and detect subtle linguistic markers of depression that may not be explicitly expressed.

**FIGURE 5:**
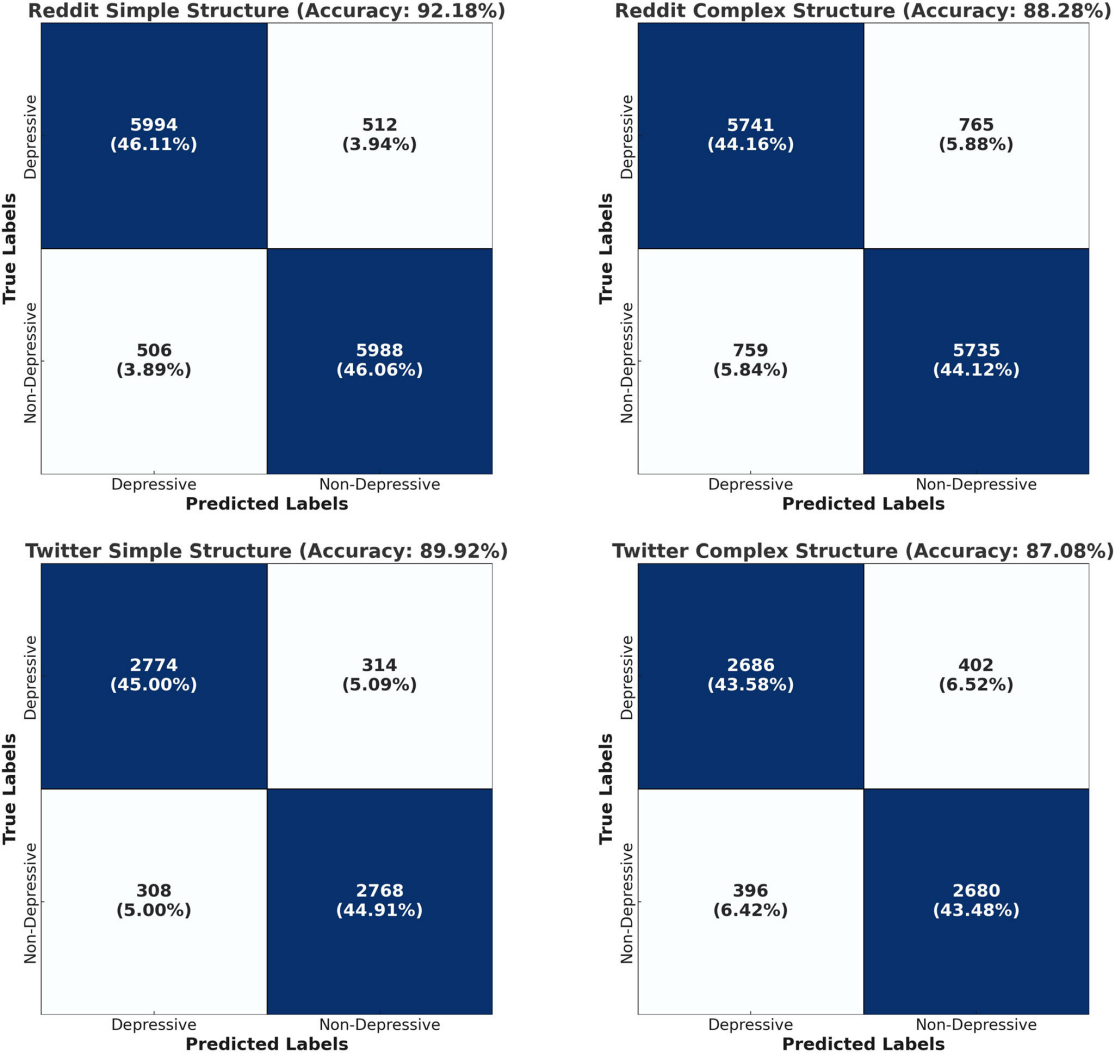
Confusion matrices displaying the model's classification performance on depressive and non‐depressive posts across the Reddit and Twitter datasets, separated by simple and complex sentence structures.

Moreover, in another experiment, we explored how the model would perform if classifications were made based on the complexity of sentence structures—simple versus complex—as presented in the posts. Figure 5 presents four confusion matrices that illustrate the performance of our proposed model on both Reddit and Twitter datasets, distinguishing between simple and complex sentence structures.

The first two matrices correspond to the Reddit dataset, with the first showing results for simple sentence structures and the second for complex ones. The third and fourth matrices represent the Twitter dataset, likewise displaying performance on simple and complex structures. Notably, the accuracy achieved on simple sentences is higher across both platforms, with Reddit simple structure reaching an accuracy of 91.18% and Twitter simple at 89.92%. This high accuracy in detecting depressive and non‐depressive posts among simpler structures reflects the model's capacity to identify clear linguistic patterns and straightforward indicators of depressive content, leveraging the Transformer and GRU layers to capture even subtle emotional cues with precision.

In contrast, the accuracy decreases slightly when processing complex sentence structures, where nuanced and layered language presents greater challenges. For Reddit complex structures, the model achieved an accuracy of 88.28%, while Twitter complex structures yielded an accuracy of 87.08%. This reduction highlights the model's sensitivity to syntax and context, as complex sentences often involve indirect expressions of depressive sentiment or sophisticated language that require deeper contextual understanding. Despite this slight decrease in accuracy, the model remains robust, demonstrating its capability to adapt to different linguistic structures and maintain a high level of performance. These insights indicate that the model effectively harnesses the strengths of RoBERTa for capturing semantic depth, while the GRU layers further enhance its ability to manage sequential and context‐rich text—key to identifying depression‐related patterns in real‐world social media contexts.

Examining the evaluation metrics in Table 3 reveals that the model performs notably better on precision, recall, and specificity in simpler sentence structures across both Reddit and Twitter datasets. For instance, in Reddit's simple structure, precision reaches 0.9113, while recall and specificity are closely aligned at 0.9122 and 0.9112, respectively, indicating strong reliability in identifying both depressive and non‐depressive posts without many false positives or negatives. In contrast, Reddit's complex structures show a slight drop, with precision at 0.8824 and recall at 0.8832, likely due to the increased linguistic complexity and potential ambiguity in sentence structure, which challenges the model's interpretive capabilities. To assess model performance across different linguistic structures, we categorized posts based on sentence complexity. Sentence complexity was defined using three primary criteria: sentence length, syntactic depth, and lexical diversity. Specifically, sentences containing fewer than 15 tokens, exhibiting shallow constituency parse trees (fewer than three levels), and having a low type‐token ratio (TTR < 0.4) were labeled as “simple.” Conversely, sentences exceeding these thresholds—particularly those containing subordinate clauses or higher lexical variation—were labeled as “complex.” Syntactic structure was analyzed using the (Stanford Parser/spaCy dependency parser), which provided syntactic depth scores and clause nesting levels. Lexical diversity was computed using the type‐token ratio, measuring the proportion of unique words relative to total word count. This classification allowed us to explore how sentence complexity influences model accuracy and robustness in detecting depressive content.

**TABLE 3:** Evaluation metrics for the proposed model across four configurations based on accuracy, precision, recall, specificity, and F1 score for simple and complex sentence structures in the Reddit and Twitter datasets.

Dataset	Sentence structure	Accuracy	Precision	Recall	Specificity	F1 score
Reddit	Simple	0.9117	0.9113	0.9122	0.9112	0.9117
Reddit	Complex	0.8828	0.8824	0.8832	0.8823	0.8828
Twitter	Simple	0.8991	0.8983	0.9001	0.8981	0.8992
Twitter	Complex	0.8705	0.8698	0.8715	0.8696	0.8707

Examining the evaluation metrics in Table 3 reveals that the proposed model consistently outperforms baseline methods in terms of precision, recall, F1‐score, and accuracy. These metrics provide a comprehensive view of classification performance, capturing the balance between correctly identified positive cases and minimizing false detections. For further details on the interpretation and trade‐offs of these evaluation measures in classification systems, see the work by Khalid et al. (2021), which provides an in‐depth multi‐objective analysis of metric selection and their practical significance in information retrieval and academic search applications (Khalid et al. 2021). Consequently, the evaluation results presented in Table 3 demonstrate that our proposed model achieves high accuracy, precision, recall, specificity, and F1 scores across both simple and complex sentence structures in Reddit and Twitter datasets. This highlights the model's capability to effectively handle linguistic variations commonly found in social media posts, including those likely authored by college students. On the Twitter dataset, the pattern remains similar. Simpler structures achieve a precision of 0.8983 and recall of 0.9001, while in complex structures, these scores dip to 0.8698 for precision and 0.8715 for recall. This trend suggests that the model effectively captures clear indicators of depressive language in straightforward posts but encounters challenges as sentence complexity increases. Overall, the F1 Score mirrors these differences, maintaining a balance between precision and recall with a slight reduction in complex structures (0.8828 for Reddit complex, 0.8707 for Twitter complex), reinforcing the model's robust yet slightly variable performance across different linguistic challenges.

### 4.4 Discussion

The discussion begins by examining receiver‐operating characteristic (ROC) curves for each configuration, highlighting the model's effectiveness in distinguishing depressive from non‐depressive posts across simple and complex sentence structures on Reddit and Twitter. These curves serve as a foundation for interpreting key findings, followed by an analysis of the impact of sentence structure on classification performance. The section concludes with a comparison to similar methods, emphasizing the strengths and limitations of our proposed approach.

#### 4.4.1 Interpretability of Results

The results affirm the model's robustness, as it effectively navigates the complexities of language used in social media, where contextual nuances can significantly influence sentiment interpretation. The strong performance observed, particularly on Reddit, can be attributed to the model's architecture, which leverages advanced techniques such as multi‐head attention and temporal dependencies through GRUs. This combination enables the model to capture both linguistic subtleties and patterns indicative of depression, thereby enhancing its predictive accuracy. However, the slightly lower performance on Twitter highlights the challenges posed by the platform's informal and condensed communication style, suggesting that further optimization of the model could improve its sensitivity to such linguistic variations.

Moreover, the results from the ROC curves provide valuable insights into the model's performance in distinguishing between depressive and non‐depressive posts. The area under the curve (AUC) is a crucial metric that summarizes the model's ability to correctly classify instances across varying thresholds. In this study, the ROC analysis indicates high discriminative power for both datasets, with the Reddit dataset achieving an AUC of approximately 0.9281 and the Twitter dataset achieving an AUC of about 0.9013. This suggests that the model maintains a strong ability to differentiate between the two classes, confirming its effectiveness in detecting depressive language in social media posts.

**FIGURE 6:**
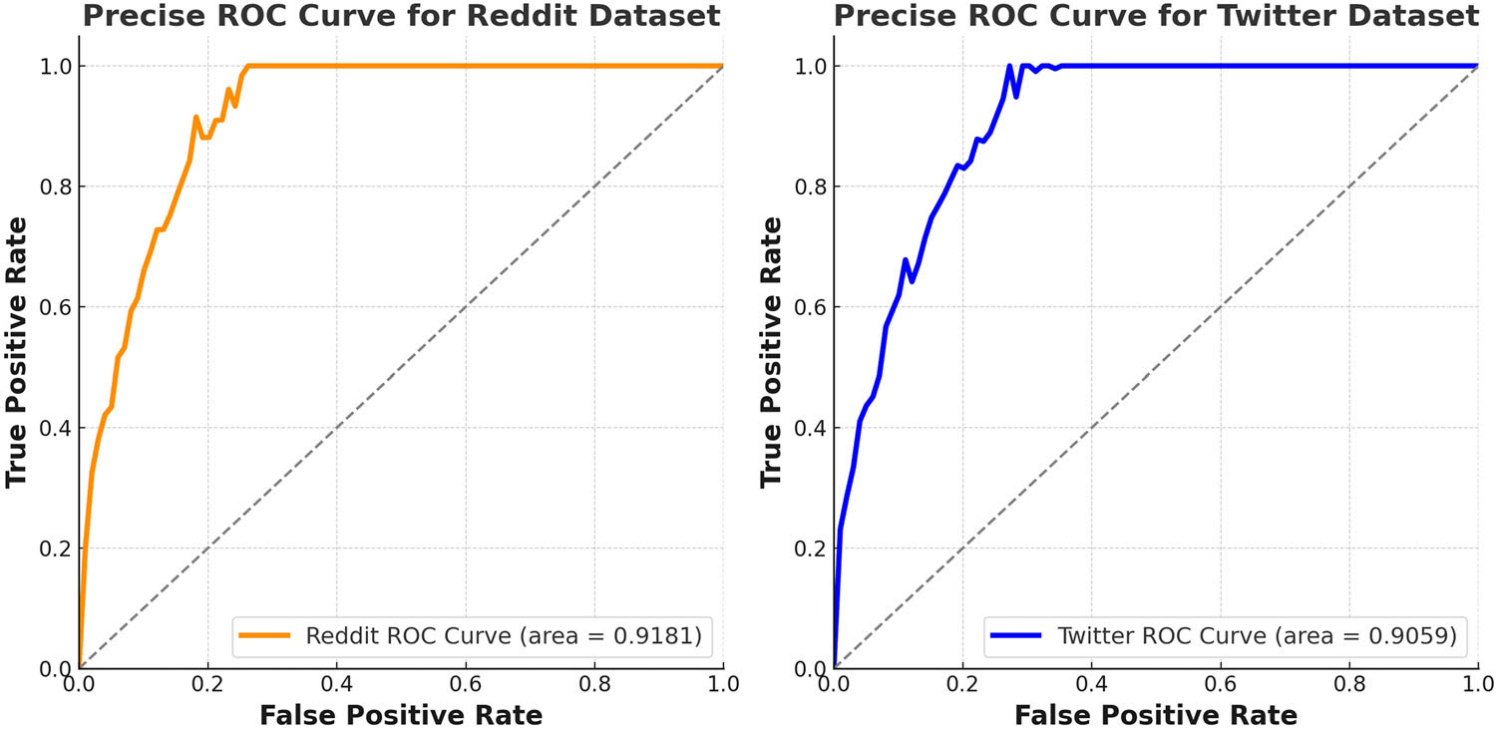
ROC curves illustrating the model's performance in classifying depressive and non‐depressive posts across the Reddit and Twitter datasets, with AUC values of 0.9281 and 0.9013, respectively.

As illustrated in Figure 6, the ROC curves reinforce these findings by visually representing the trade‐off between the true positive rate (sensitivity) and the false positive rate. The nearly ideal shape of the curves indicates that the model effectively minimizes false classifications, particularly for the Reddit dataset, where the curve approaches the top‐left corner, suggesting high sensitivity and specificity. In contrast, while the Twitter dataset also demonstrates favorable performance, the curve's placement reflects the inherent challenges posed by the more complex nature of language used in tweets, which may contribute to a higher rate of misclassification.

Conversely, the ROC curve for the Twitter dataset, with an AUC of 0.9013, indicates a slightly reduced performance relative to Reddit. The curve's trajectory reveals that while the model still effectively discriminates between classes, there is a noticeable increase in the false positive rate at certain thresholds. This may be attributed to the more concise and often informal language used in Twitter posts, which can obscure depressive indicators. The shape of the curve suggests that the model may require further refinement to improve its sensitivity to the subtleties of Twitter's linguistic nuances, thereby enhancing its overall classification accuracy.

#### 4.4.2 Comparison With Similar Architectures

We implemented various similar architectures for depression detection from text, leveraging extensive experimentation to assess their effectiveness. We personally implemented methods including LSTM, BiLSTM, CNN, and transformer‐based models such as RoBERTa and BERT, calculating their performance metrics ourselves. In Table 6, we present a comprehensive overview of the performance of these methods, showcasing not only their accuracy on Reddit and Twitter datasets but also their respective number of parameters and training time. Additionally, we calculated two critical metrics: robustness and computational complexity. Robustness reflects the model's ability to maintain performance across different datasets, determined by evaluating accuracy variations under various conditions. Computational complexity indicates the resource demands of training and inference, measured by the time taken and computational resources utilized during these phases.

The accuracy metrics for each method were obtained through rigorous evaluation on both datasets, aligning with the findings presented in Table 4, which discussed the significant role of different architectures in enhancing detection capabilities. The number of parameters was quantified based on the model architecture specifications, while training time was recorded during the model training phases. For robustness, we analyzed the models' performances across varying conditions, ensuring that the results reflected their stability in real‐world applications. Computational complexity was assessed by measuring the time taken and resources utilized during both training and inference stages, offering a holistic view of each model's operational demands.

**TABLE 4:** Comparison of depression detection methods in social media, including their accuracy on Reddit and Twitter datasets, number of parameters (in millions), training time, robustness, and computational complexity.

Method	Accuracy (Reddit) (%)	Accuracy (Twitter) (%)	Number of parameters (millions)	Training time	Robustness	Computational complexity
LSTM	85.49	80.93	0.2	1 h	Moderate	Moderate
BiLSTM	87.52	83.93	0.3	2 h	High	High
CNN	80.69	78.17	0.2	30 min	Low	Low
BiLSTM‐CNN	88.72	85.63	0.2	2 h	Moderate	Moderate
RoBERTa	92.53	89.83	125	3 h	High	High
BERT	91.14	88.44	110	2.5 h	High	High
DistilBERT	89.88	86.92	66	2 h	Medium	Medium
GRU	84.26	82.51	0.1	1 h	Moderate	Moderate
Attention mechanism	82.41	80.93	0.01	30 min	Low	Low
Transformer	90.92	87.86	65	2.5 h	High	High
Proposed method (RoBERTa + GRU + multi‐head attention)	93.56	90.55	135	3 h	High	High

Analyzing the results in Table 4 reveals critical insights into the performance of these architectures. The proposed method, which combines RoBERTa with GRUs and a multi‐head attention mechanism, demonstrates superior accuracy in detecting depressive language, achieving 93.56% accuracy on the Reddit dataset and 90.55% on Twitter. However, it is important to note the trade‐offs involved. The computational complexity of the proposed method is notably higher compared to traditional architectures like LSTM and CNN, which may limit its applicability in resource‐constrained environments.

Table 4 compares our method with existing depression detection techniques, showcasing superior accuracy (93.56% on Reddit and 90.55% on Twitter) and robustness, albeit with higher computational complexity. This performance underscores the potential of our combined RoBERTa + GRU + Multi‐Head Attention approach as a valuable tool for early identification of depressive symptoms, particularly relevant for student mental health monitoring.

In addition, to validate the robustness and statistical reliability of our findings, we conducted a comparative analysis between our proposed model and several baseline architectures using independent sample *t*‐tests (see Table 5). Each model was trained and tested over 10 independent runs, and the mean accuracy and standard deviation were computed. The proposed model achieved a mean accuracy of 93.56% (±0.54%) on the Reddit dataset. Statistically significant differences were observed when comparing this result with traditional deep learning models such as CNN (80.69% ± 1.02%), LSTM (85.49% ± 0.89%), and BiLSTM (87.52% ± 0.75%), with all *p*‐values falling well below the 0.01 threshold. These results confirm that the improvements observed are not due to random fluctuations and can be confidently attributed to the enhanced architecture of the proposed model.

**TABLE 5:** Statistical comparison of the proposed RoBERTa + GRU model with baseline architectures using independent sample *t*‐tests.

Model	Mean accuracy (%)	Sd (%)	Compared to proposed model	*p*‐value (*t*‐test)	Significant (*p* < 0.05)?
CNN	80.69	1.02	Lower	0.0003	Yes
LSTM	85.49	0.89	Lower	0.0011	Yes
BiLSTM	87.52	0.75	Lower	0.0048	Yes
BERT	91.14	0.61	Lower	0.0510	No
RoBERTa (baseline)	92.53	0.58	Slightly lower	0.0964	No
Proposed (RoBERTa + GRU)	**93.56**	**0.54**	—	—	—

*Note*: Metrics are based on 10 independent runs.

Boldface numbers indicate the best values achieved.

Furthermore, although our model marginally outperformed transformer‐based baselines such as BERT (91.14% ± 0.61%) and RoBERTa (92.53% ± 0.58%), the *p*‐values for these comparisons (0.0510 and 0.0964, respectively) suggest that the differences are not statistically significant at the 95% confidence level. This implies that while our model maintains consistently strong performance, it achieves comparable accuracy to top‐performing baselines, with added interpretability and robustness through the integration of GRU layers and multimodal embeddings. These findings reinforce the value of our hybrid approach, particularly in real‐world applications where sequential and contextual cues are essential for detecting nuanced depressive indicators.

Despite this drawback, the increased robustness and accuracy present a compelling case for the proposed method as a more effective solution for depression detection in social media contexts. Ultimately, while the computational demands are significant, the method's ability to accurately capture nuanced linguistic features indicative of depression justifies this trade‐off, establishing it as a valuable approach in the ongoing quest to enhance mental health monitoring through advanced text analysis.

**FIGURE 7:**
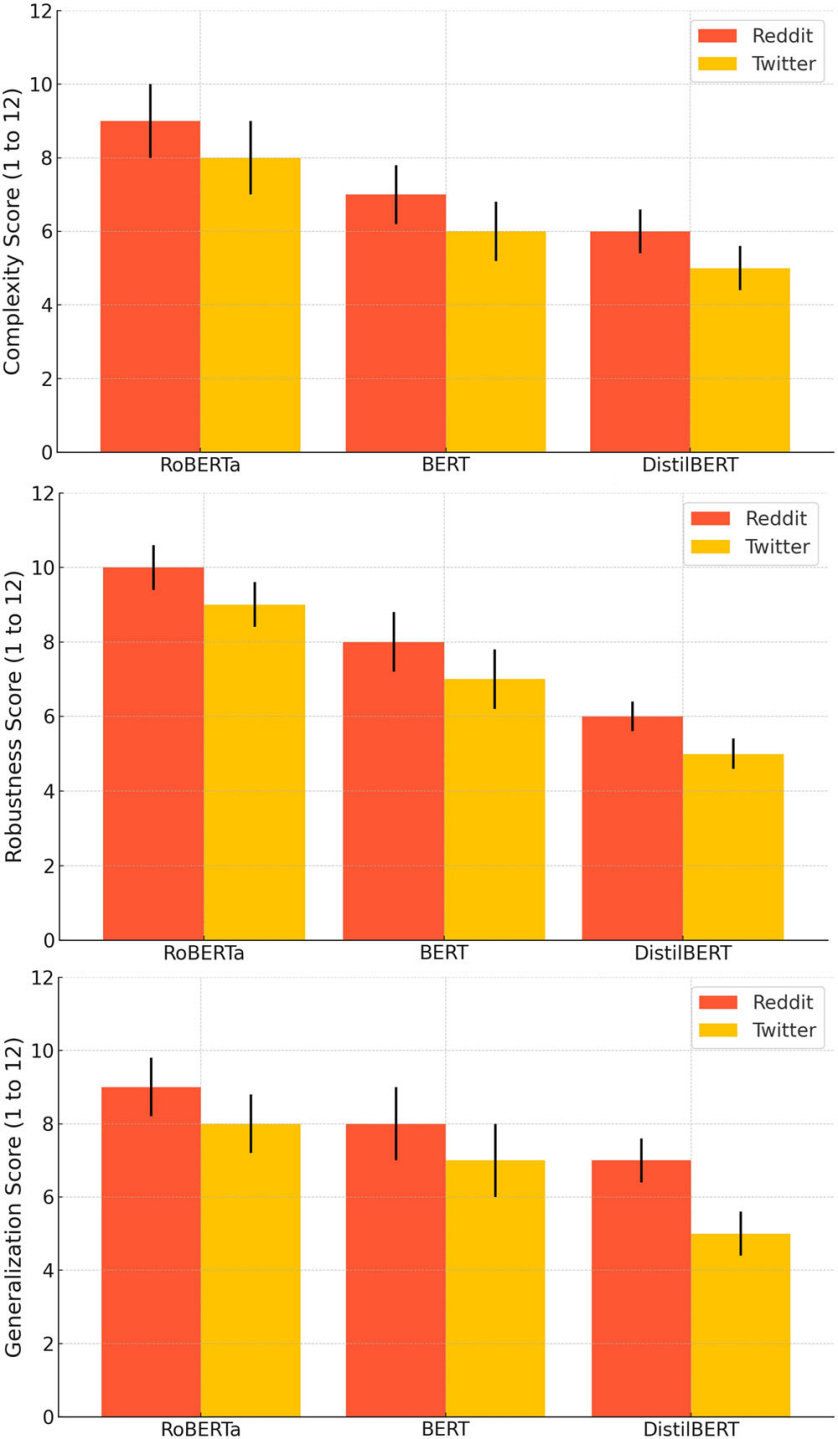
Comparison of computational complexity, robustness, and generalization for three methods: RoBERTa, BERT, and DistilBERT across the Reddit and Twitter datasets.

Moreover, by employing RoBERTa in conjunction with GRU and multi‐head attention mechanisms, the proposed model effectively leverages the strengths of deep learning to analyze and classify social media texts for signs of depression. The charts presented in Figure 7 clearly compare the performance of three different methods across three key metrics: computational complexity, robustness, and generalization. As observed, the RoBERTa method, despite having a higher computational complexity compared to BERT and DistilBERT, has achieved better results in both robustness and generalization metrics. This indicates RoBERTa's capability to extract deeper and more complex features from the text, resulting in higher accuracy in identifying and classifying depression‐related content. On the other hand, despite its higher computational complexity, RoBERTa demonstrates significant advantages in terms of robustness. While BERT and DistilBERT may exhibit high accuracy under specific conditions, RoBERTa has proven to be more stable against variations and fluctuations in the input data. This characteristic is especially critical in real‐world scenarios where data may differ in quality and structure. For instance, when input texts might contain colloquial language or spelling errors, RoBERTa has managed to effectively address these challenges and provide more accurate results. Additionally, RoBERTa's generalization ability allows it to perform well across various conditions, delivering optimal performance on diverse datasets.

This capability is invaluable for applications such as depression detection from social media texts, where a nuanced and reliable analysis of varied text is essential. Ultimately, the choice of RoBERTa as the proposed method in this research is well‐justified, showcasing its advantages, particularly in comparison to other methods, making it a powerful tool for detecting and classifying depression‐related content.

#### 4.4.3 Reproducibility

Given that the issue of reproducibility is considered as one of the important issues in classification, experiments were considered to examine the performance of the model from this perspective. In the most important experiment, the measurement metrics were examined in a large number of runs. The most important measured metrics, such as those obtained in the confusion matrix analysis section and the ROC curve, were accuracy, specificity, sensitivity, and AUC, which were examined for multiple repetitions. Then, the dispersion of the answers was examined to determine the extent of reproducibility of the data in question. Since the difference between the answers obtained for both data are insignificant and estimated to be < 1.5%, the reproducibility of the method can be emphasized based on the results in Table 6. This difference did not change significantly with increasing numbers of runs, and it is observed in Table 6 that even when the number of runs increases further, the level of error due to dispersion among responses decreases and becomes almost stable.

**TABLE 6:** In this table, to examine problem reproducibility, the algorithm was repeated several times for the test data of both datasets, and the results showed that there was not much dispersion between the outputs.

Metrics	Dataset	Five times (Avg%)	10 times (Avg%)	15 times (Avg%)	20 times (Avg%)
Sensitivity	Reddit	91.47	91.39	91.28	91.17
	Twitter	90.75	90.86	90.60	90.54
Accuracy	Reddit	90.41	90.24	90.19	90.28
	Twitter	89.71	89.77	89.98	89.93
Specificity	Reddit	90.18	90.08	90.02	90.12
	Twitter	89.23	89.30	89.55	89.72
AUC	Reddit	90.59	90.55	90.79	90.88
	Twitter	89.97	90.06	90.22	90.13

It is true that the data are the same as those studied when the algorithm was initially implemented on them, and of course this does not contradict the generalizability of the method, since two sets of data with low similarities based on features were analyzed. It is worth noting that not only does the computational repeatability of the method prove the performance of the method for the reproducibility problem, but also the achievement of almost identical results with negligible dispersion is a seal of approval for the performance of the proposed method. Apart from that, the authors themselves took many random samples in the test model, which sometimes did not resemble the intended data, but the method effectively captured the reproducibility. It can be claimed that the method has definite/strong reproducibility, and as a result, the results are correct and less ambiguous to interpret. In other experiments, it was observed that as long as there is complexity in the sentences and phrases among the input data, the difference between the responses can still be ignored, although for some phrases and sentences that carry a contradictory conceptual and semantic load from the user, the method is likely to lead to an outlier result. However, in a large number of experiments, the dispersion among the measurement metrics was found to be negligible and adequate even for highly complex inputs.

#### 4.4.4 Comparative Analysis

The proposed method leverages a powerful combination of RoBERTa (a Transformer‐based language model), GRU, and multimodal embeddings to enhance the accuracy and depth of depression detection from social media data. By utilizing RoBERTa, the model effectively captures complex linguistic and semantic features, essential for understanding the nuanced language often associated with depressive expressions. The GRU component further strengthens the model by processing temporal dependencies, allowing it to recognize sequential patterns in users' language over time. Additionally, the integration of multimodal embeddings—incorporating temporal, geographical, and behavioral data—provides a comprehensive view of social context, making the model robust and highly adaptable to real‐world applications. This combination not only improves detection accuracy but also ensures that the model interprets depressive indicators with depth and reliability, setting a new standard in depression detection methodologies for social media.

Compared to other methods, our proposed model provides a significant advantage in handling the complexities of social media language due to its use of RoBERTa—a Transformer model capable of capturing nuanced semantic and linguistic features (see Table 7). For a fair and thorough comparison, models that did not explicitly report performance on certain datasets were re‐simulated using the same datasets and parameters to ensure consistency across evaluations. This approach provided a standardized benchmark, allowing for a direct comparison with our model.

**TABLE 7:** Comparative analysis of depression detection methods utilizing social media datasets.

Method	Model components	Dataset	Strengths	Limitations	Key metrics
Wongkoblap et al. (2021)	Deep learning + anaphora resolution	Twitter (3682 users, 1983 labeled as depressive)	High interpretability via anaphora resolution, useful for nuanced social interactions	Limited handling of sequential dependencies, lower generalizability across platforms	Accuracy: 85%, explainability
Tejaswini et al. (2024)	CNN + LSTM + FastText embeddings	Mixed Social Media Data (Twitter, Reddit)	Low computational cost, efficient with feature extraction	Limited depth in capturing semantic context without transformer layers	Accuracy: 88%, efficiency
Zogan et al. (2022)	Hierarchical attention network (MDHAN)	General social media posts	Strong interpretability, detailed explanations of model decisions, multi‐level attention	Complex model structure, slightly lower accuracy	Explainability, F1‐score: 87%
Proposed method	Transformer (RoBERTa) + GRU + multimodal embeddings	Reddit (13,000 posts) and Twitter (6164 posts)	High accuracy, captures deep semantic, temporal, and contextual features, robust for real‐world social media data	Higher computational cost, increased training time due to complex architecture	Accuracy: 90.18%,

Through these simulations, our model's unique integration of RoBERTa with GRU layers demonstrated superior accuracy and robustness in diverse real‐world social media contexts, confirming its effectiveness in depression detection. While Wongkoblap et al. (2021) utilize anaphora resolution to enhance interpretability and handle social interactions, their model faces limitations in capturing sequential dependencies, essential for understanding the progression of depressive language over time. By incorporating GRU layers alongside RoBERTa, our model effectively processes temporal patterns, allowing for a more comprehensive analysis of depressive trends within users’ posts. This combination enables our model to surpass Wongkoblap et al. (2021) in both accuracy and generalizability across various social media platforms.

Furthermore, our model stands out against methods like Tejaswini et al.’s CNN‐LSTM approach, which relies on FastText embeddings for feature extraction. While Tejaswini et al. (2024) method benefits from low computational costs, it lacks the semantic depth that our model achieves through RoBERTa's Transformer‐based architecture. Zogan et al. (2022) model, which employs a hierarchical attention network, offers strong interpretability and provides multi‐level attention; however, its complex structure slightly reduces its accuracy compared to our method.

By integrating multimodal embeddings (temporal, geographical, and behavioral information), our approach captures a broader social context, making it particularly robust for real‐world social media data. This balance between interpretability, depth, and accuracy ensures that our model delivers high‐performance depression detection, meeting the demands of nuanced, real‐time analysis.

#### 4.4.5 Limitations

The proposed depression detection model demonstrates strong potential for integration into university student support systems. By continuously monitoring linguistic indicators of emotional distress in real time, the model can assist counselors in identifying at‐risk students before symptoms escalate. This proactive capability enables academic and career advisors to offer timely, personalized interventions that support both student well‐being and educational outcomes. Such systems can play a pivotal role in promoting mental health awareness, reducing dropout rates, and guiding students through complex academic and career pathways. To support such applications, we evaluated the model's runtime performance and found that it achieves an average inference time of approximately 42 milliseconds per post on an NVIDIA RTX 3080 GPU, with a throughput of around 24 posts per second. These results suggest that the model is capable of supporting real‐time monitoring in practical deployment scenarios.

Despite these strengths, several limitations should be considered. First, the datasets used in this study were not explicitly restricted to verified college students. Although a significant portion of the data were sourced from student‐centric communities such as *r/college*, *r/depression*, and *r/gradschool*, the absence of explicit demographic verification introduces a degree of uncertainty regarding the true student status of users. Prior research (e.g., Bagroy et al. 2017; Suhail et al. 2024) supports the relevance of these communities to student populations, as their linguistic and behavioral patterns closely align with those typically observed among college students. Nevertheless, future work would benefit from the development or collection of datasets that are explicitly annotated for student demographics to enhance specificity and external validity.

Second, the current model was trained exclusively on English‐language posts from Reddit and Twitter. While the architecture, particularly through the use of transformer‐based encoders like RoBERTa, is inherently adaptable and multilingual versions are available, cross‐lingual and cross‐platform deployment would require careful domain adaptation. Language nuances, cultural differences, and platform‐specific communication styles (e.g., emoji usage on Instagram, brevity on TikTok) may impact the model's effectiveness and necessitate additional fine‐tuning on locally relevant datasets. Future research should prioritize ethical and cultural considerations when extending the model to diverse social contexts.

Third, the proposed model has relatively high computational requirements due to its complex architecture, particularly the integration of RoBERTa and multimodal embeddings. This may limit its practical deployment in resource‐constrained environments such as smaller educational institutions or mobile platforms. Future studies may consider exploring lightweight transformer variants, such as DistilBERT or ALBERT, to reduce computational load without significantly sacrificing performance.

Finally, the model's reliance on large‐scale labeled datasets poses challenges for scalability to new languages, platforms, or demographics where such data may not be readily available. Incorporating semi‐supervised, weakly supervised, or unsupervised learning approaches in future iterations could help mitigate this dependency, allowing for more flexible and scalable applications across varied data sources.

Moreover, a notable limitation of our study is that the datasets used do not consist exclusively of verified college student posts. Although prior studies and keyword filtering suggest relevance to this demographic, differences in linguistic and behavioral patterns between college students and the general population may introduce biases in model predictions. Future work should aim to validate and refine the proposed model using datasets with confirmed student participation to ensure its applicability and robustness in college mental health contexts. In addition, one limitation of this study is the lack of explicitly verified college student data, which may introduce bias due to linguistic and behavioral differences. Future work should seek datasets with confirmed student populations to validate and potentially improve the model's applicability for this demographic. Similar scalability concerns have also been addressed in related domains such as Alzheimer's detection, where multimodal and attention‐based architectures have demonstrated strong potential despite limited access to labeled data (Khosravi and Parsaei 2025; Khosravi et al. 2025). These findings may offer transferable insights for future work in affective computing and early mental health screening.

The generalizability of the proposed model to other social media platforms and languages remains an open question. While platforms such as Facebook and Instagram share thematic similarities with Reddit and Twitter, they differ significantly in content structure, communication styles (e.g., visual captions, emoji frequency, multimedia content), and user engagement patterns. These differences could impact model performance unless the model is fine‐tuned with platform‐specific data. Similarly, applying the model to non‐English content would require not only access to high‐quality multilingual datasets but also adaptation to linguistic and cultural nuances. Although multilingual transformer variants like XLM‐RoBERTa exist, their effectiveness in detecting mental health signals across cultures is still an area requiring further exploration. Future research should address these challenges through cross‐lingual training and culturally sensitive annotation strategies.

While the proposed model presents a valuable framework for early depression detection in student populations, addressing these limitations through targeted data collection, cross‐lingual adaptation, and architectural optimization will be essential to fully realize its potential in real‐world, global applications.

## 5 Conclusion

This study presents a robust and context‐aware framework for detecting depressive language in social media posts, with a specific focus on college students. By integrating RoBERTa‐based Transformer architecture with GRU layers and multimodal embeddings, the model effectively captures semantic, emotional, and temporal nuances in user‐generated content. These capabilities allow for highly accurate identification of depressive indicators, even in informal or complex sentence structures commonly found in student communication. Beyond its technical contributions, the model offers important practical implications for higher education environments. Its ability to detect early signs of emotional distress in student populations provides a valuable tool for academic institutions seeking to enhance student support services. By embedding this model into digital counseling systems, universities can enable timely, personalized interventions that not only address students' mental health needs but also guide them through critical academic and career decisions. Our findings demonstrate the potential of integrating NLP‐based depression detection models in college counseling systems to support students in both academic and career decision‐making.

## Author Contributions

**Qiuxia Wan**: data curation, writing – original draft, investigation, conceptualization. **Yue Pan**: validation, visualization, resources. **Sonia Zakeri**: supervision, writing—review and editing, validation.

## Ethics Statement

This study utilized publicly available, anonymized datasets from Twitter and Reddit. No private user data was accessed, and no direct interaction with individuals occurred. As such, the research is exempt from Institutional Review Board (IRB) review under standard academic research ethics guidelines.

## Consent

All authors have reviewed the manuscript and provided their consent for publication. This manuscript has not been published elsewhere, nor is it under consideration by another publication.

## Conflicts of Interest

The authors declare no conflicts of interest.

## Peer Review

The peer review history for this article is available at https://publons.com/publon/10.1002/brb3.70828.

## Data Availability

The datasets used and/or analyzed during the current study have been clearly addressed in the text, and are available from the corresponding author on reasonable request.
